# Quantitative validation of Monte Carlo SPECT simulation: application to a Mediso AnyScan GATE simulation

**DOI:** 10.1186/s40658-023-00581-4

**Published:** 2023-09-30

**Authors:** Sophia Pells, David M. Cullen, Daniel Deidda, Ana M. Denis-Bacelar, Andrew Fenwick, Kelley M. Ferreira, David Hamilton, Warda Heetun, Peter Julyan, George Needham, Ben Pietras, Emlyn Price, James Scuffham, Jill Tipping, Andrew P. Robinson

**Affiliations:** 1 Department of Physics and Astronomy, The University of Manchester, Manchester, UK; 2 National Physical Laboratory, Teddington, UK; 3 Department of Radiology, UMass Chan Medical School, Worcester, MA USA; 4 The Christie NHS Foundation Trust, Manchester, UK; 5 Royal Surrey County Hospital, Guildford, UK

**Keywords:** Monte Carlo, GATE, Validation, SPECT, Quantification

## Abstract

**Background:**

Monte Carlo (MC) simulations are used in nuclear medicine imaging as they provide unparalleled insight into processes that are not directly experimentally measurable, such as scatter and attenuation in an acquisition. Whilst MC is often used to provide a ‘ground-truth’, this is only the case if the simulation is fully validated against experimental data. This work presents a quantitative validation for a MC simulation of a single-photon emission computed tomography (SPECT) system.

**Methods:**

An MC simulation model of the Mediso AnyScan SCP SPECT system installed at the UK National Physical Laboratory was developed in the GATE (Geant4 Application for Tomographic Emission) toolkit. Components of the detector head and two collimator configurations were modelled according to technical specifications and physical measurements. Experimental detection efficiency measurements were collected for a range of energies, permitting an energy-dependent intrinsic camera efficiency correction function to be determined and applied to the simulation on an event-by-event basis. Experimental data were collected in a range of geometries with $^{99\text {m}}$Tc for comparison to simulation. The procedure was then repeated with $^{177}$Lu to determine how the validation extended to another isotope and set of collimators.

**Results:**

The simulation’s spatial resolution, sensitivity, energy spectra and the projection images were compared with experimental measurements. The simulation and experimental uncertainties were determined and propagated to all calculations, permitting the quantitative agreement between simulated and experimental SPECT acquisitions to be determined. Statistical agreement was seen in sinograms and projection images of both $^{99\text {m}}$Tc and $^{177}$Lu data. Average simulated and experimental sensitivity ratios of ($0.991 \pm 0.011$) were seen for emission and scatter windows of $^{99\text {m}}$Tc, and ($0.897 \pm 0.014$) and ($0.839 \pm 0.014$) for the 113 and 208 keV emissions of $^{177}$Lu, respectively.

**Conclusions:**

MC simulations will always be an approximation of a physical system and the level of agreement should be assessed. A validation method is presented to quantify the level of agreement between a simulation model and a physical SPECT system.

## Background

The application of Monte Carlo (MC) simulation in nuclear medicine imaging has a long history. It has been widely used in relation to single-photon emission computed tomography (SPECT) imaging, from early studies of Compton scatter [1], to establishing practical methods for scatter correction [2], and work modelling full SPECT systems and collimator models [3–5]. Multiple MC packages are available for nuclear medicine applications [6–10].

To ensure that results generated from a simulation (MC or other method) are adequate for their intended use, the simulation must be validated. This should be achieved through comparison of experimental and simulated data with known measurement uncertainties [11]. Full validation of a MC SPECT simulation requires validation of the emission, transport and interaction of radiation, the detection system response, and validation that the generated tomographic information is comparable to the physical camera’s response. Validation therefore requires consideration of a number of experimental observables including the energy spectra, spatial resolution, sensitivity, movement of the gamma camera heads during the acquisition and the recorded sinogram (counts per time frame). If the projection images are to be reconstructed, additional validation of the reconstruction process is required. In this work, we present a quantitative validation of a SPECT MC simulation by comparison to experimental measurements.

This work used the GATE (Geant4 Application for Tomographic Emission) package [12], an open-source extension to the Geant4 toolkit [13] dedicated to MC in medical physics. GATE models have been developed for many different SPECT systems; a summary of publications can be found in [5]. All GATE model parameters, such as detector geometry and physics processes, are configured by the user in GATE macros. It is then up to the user to validate their simulation model with comparison to experimental data. Many internal components of SPECT cameras, such as the electronics and collimator internal geometries, cannot be readily experimentally measured by the user. Therefore, simulation models often rely on manufacturer-provided technical specifications for the geometry and composition of system components. Due to manufacturing tolerances, there may be differences between these specifications and the manufactured systems, leading to differences in photon interactions specific to each system. Thus, experimental data must be acquired to validate the simulation for each system independently. For simulations of SPECT systems, an accurate collimator model is particularly important, so the photon interactions with the collimator (such as septal penetration, scatter and X-ray fluorescence) are modelled. Realistic parallel hole collimators have complex geometries with tens to hundreds of thousands of holes which can lead to long computation times for MC simulations [14].

An MC model of the Mediso AnyScan SCP system installed at the UK National Physical Laboratory (NPL) was developed in the GATE framework, and a validation methodology was tested. To date, there are no published works of full MC simulations of this system (to the authors’ knowledge). The focus of this validation methodology was on quantifying experimental and simulation uncertainties and evaluating the agreement between the simulation and experiment within their context. This quantitative validation methodology would be applicable to a simulation of any camera in any MC software.

This validation procedure was performed for the commonly used clinical isotope $^{99\text {m}}$Tc. Planar acquisitions of point sources and SPECT imaging of water-filled phantoms were used. The same simulation was then tested with phantoms of $^{177}$Lu, to determine how the validation translated to another isotope and a different set of collimators.

## Methods

**Fig. 1:**
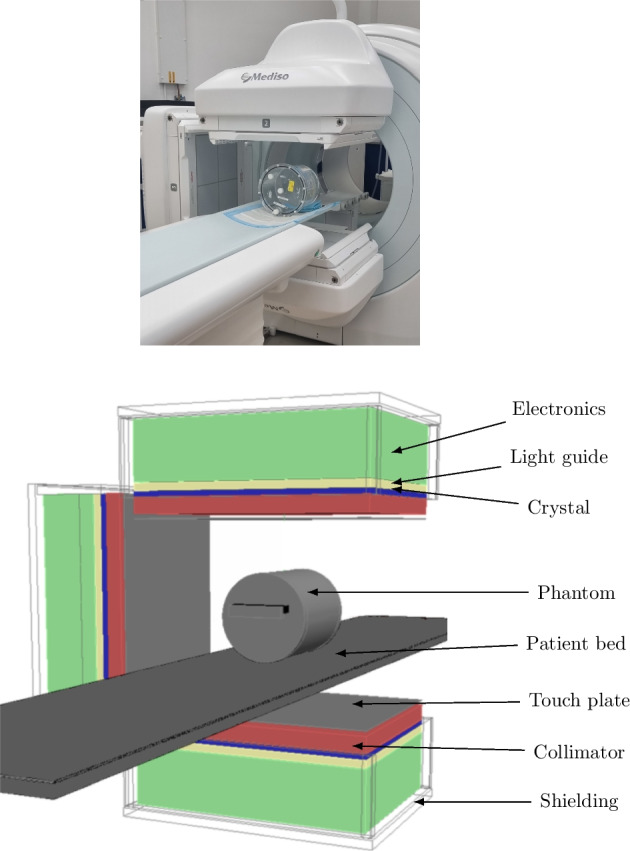
Top: A photograph of the SPECT system in dual head configuration. The third head (left) is not used to acquire data in this mode. Bottom: A visualisation of the simulation geometry with key components labelled. Each detector head is surrounded by lead shielding; this is shown as a wire frame here but fully encases each head

The Mediso AnyScan Trio SCP (SPECT-CT-PET) system is a hybrid imaging system with three imaging modalities. The SPECT component of the model installed at NPL has a NaI(Tl) scintillation crystal thickness of 9.5 mm and three detector heads. It can operate either in triple- or dual-head acquisition mode where the detector heads are at a 120$^\circ$ or 180$^\circ$ separation, respectively. In dual-head acquisition mode, the third head is placed at 90$^\circ$ to the other two, but does not acquire counts. A photograph of the system in dual-head acquisition mode is shown in Fig. 1. Two collimators were considered in this work: Low Energy High Resolution (LEHR) and Medium-Low Energy General Purpose (MLEGP) collimators, recommended for imaging with $^{99\text {m}}$Tc and $^{177}$Lu, respectively. A description of their hole sizes and septal thicknesses from the Mediso AnyScan catalogue [15] is given in Table 1.

**Table 1:** The specifications of the low energy high-resolution (LEHR) and medium-low energy general purpose (MLEGP) collimators used in this work

Collimator	LEHR	MLEGP
Hole length (mm)	35	28
Hole diameter (mm)	1.5	1.9
Septal thickness (mm)	0.16	0.5

### Experimental data acquisition

#### Phantom acquisitions

Experimental acquisitions of $^{99\text {m}}$Tc and $^{177}$Lu were performed with LEHR and MLEGP collimators, respectively. Images of each isotope were acquired with a uniform and a localised distribution of activity. A cylinder with 20 cm internal diameter and height was filled with a uniform solution of $^{99\text {m}}$Tc. A commercial cylindrical Jaszczak phantom was used for the $^{177}$Lu acquisition; with an internal height of 18.6 cm and a diameter of 21.6 cm [16]. A commercial NEMA phantom [17] with six active spheres in an inactive background of water was used for both isotopes. The same activity concentration was dispensed to all spheres. Water was used as a scattering medium due to its similar density to human tissue. All phantom scans were performed in dual-head acquisition mode with a fixed detector radius of 250 mm; they are summarised in Table 2.

One emission (EM) and one scatter (SC) windows were simultaneously acquired for $^{99\text {m}}$Tc imaging. A larger (5%) scatter window was used for the cylindrical phantom. 20% emission and 6% scatter windows were acquired for the two emissions of $^{177}$Lu [18, 19]. The Mediso AnyScan system permits a maximum of four windows per scan so two consecutive scans with one emission and the adjacent scatter windows were performed.

**Table 2:** Details of the phantom scans performed

Isotope	Phantom	Activity (MBq)	Time per projection (s)
$^{99\text {m}}$Tc	Cylinder	103.91 ± 0.93	20
$^{99\text {m}}$Tc	6-sphere NEMA	112.9 ± 1.0	32
$^{177}$Lu	Cylinder	324.3 ± 1.6	32
$^{177}$Lu	6-sphere NEMA	173.52 ± 0.87	32

Energy window	Range (keV)	Energy window	Range (keV)
^99m^Tc		^177^Lu	
EM	140.5 ± 10%	EM1	112.9 ± 10%
SC (Cylinder)	119.0 ± 5%	SC1	98.7 ± 3%
SC (NEMA)	122.5 ± 3%	SC2	128.1 ± 3%
		EM2	208.4 ± 10%
		SC3	182.1 ± 3%
		SC4	236.3 ± 3%

Each acquisition was dual-head and tomographic, with the two SPECT heads each rotating over 180$^\circ$ in 60 projection positions at fixed radius. The activity in the phantom at the start of each SPECT acquisition is provided (the activity at the start of the EM1 acquisition is given for $^{177}$Lu). The total activity in all spheres is given for the NEMA phantoms. The energy windows used in each scan are also given; EM refers to a photopeak emission window and SC to a scatter window

Source activities were measured with the NPL secondary standard ionisation chamber system (Vinten 671). The uncertainty budgets for the NEMA phantom activities are given in Table 3. A final uncertainty of 0.892% and 0.503% was obtained, through adding the components in quadrature, for $^{99\text {m}}$Tc and $^{177}$Lu, respectively.

**Table 3:** The percentage uncertainty components for activity per unit mass of $^{99\text {m}}$Tc and $^{177}$Lu solutions used in the NEMA phantom

Uncertainty component	Uncertainty (%)	
	^99m^Tc	^177^Lu
Capacitance	0.1	0.1
Ion chamber reproducibility	0.1	0.1
Calibration factor	0.88	0.48
Standard error of the mean	0.0104	0.0209
Voltage change measurement	0.00624	0.00441
Volume correction	0.00061	0.00007
Decay correction to reference time	0.01074	0.00039
Decay correction within measurement (start to middle)	0.00326	0.00013
Decay correction within measurement (end to middle)	0.00326	0.00013
Background	0.01139	0.04539
Weighing (negligible)	1.51 $\times 10^{-9}$	3.02 $\times 10^{-9}$
Combined uncertainty	0.892	0.503

All components were combined in quadrature

All experimental images were acquired in DICOM format with 128$\times$128 matrices and voxel sizes of (4.2578 mm)$^3$. A CT scan was performed directly following each SPECT scan, with 512$\times$512 matrices of $0.9766 \times 0.9766 \times 0.625$ mm$^3$ voxels.

#### Extrinsic spatial resolution

Point source measurements of $^{99\text {m}}$Tc were obtained to measure the extrinsic spatial resolution of the system with LEHR collimators. An active volume of approximately 0.5 ml and 12 MBq was drawn into a 2.5 mL syringe which was positioned in a central, reproducible position on a custom-made perspex board. A single detector head was positioned directly above the patient bed, at the minimum height permitted by the system (106.5 mm). A static planar acquisition was conducted with a termination condition of 500,000 counts in an energy window of 140.5 keV ± 10%. This process was repeated for three additional head radii: 150, 250 and 350 mm. Further measurements were made with the head at 106.5 mm height and the source shifted laterally (150 mm in the long axis of SPECT head - ‘CentreLeft’ and −150 mm in the long axis and 100 mm in the short axis - ‘TopRight’). All experimental acquisitions used a 1024$\times$1024 matrix with pixel width 0.5322 mm to reduce the uncertainty on experimental position in the scan (half the acquisition pixel size, or 0.2661 mm). The experimental acquisitions were then down-sampled to 128$\times$128 matrices to match SPECT pixel size used in the rest of this work.

#### Background radiation

**Fig. 2:**
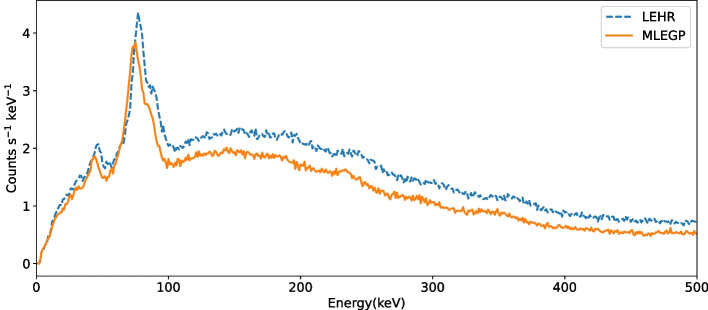
Experimental energy spectra for the background acquisitions with LEHR and MLEGP collimators. The spectra have been plotted with 1 keV bins

Extrinsic background acquisitions with LEHR and MLEGP collimators were performed to record counts with no source present. Tomographic acquisitions were acquired for all energy windows used in the phantom acquisitions for 32 s per projection. The energy spectra were extracted directly using manufacturer-provided software and are shown in Fig. 2.

#### Intrinsic camera efficiency

**Fig. 3:**
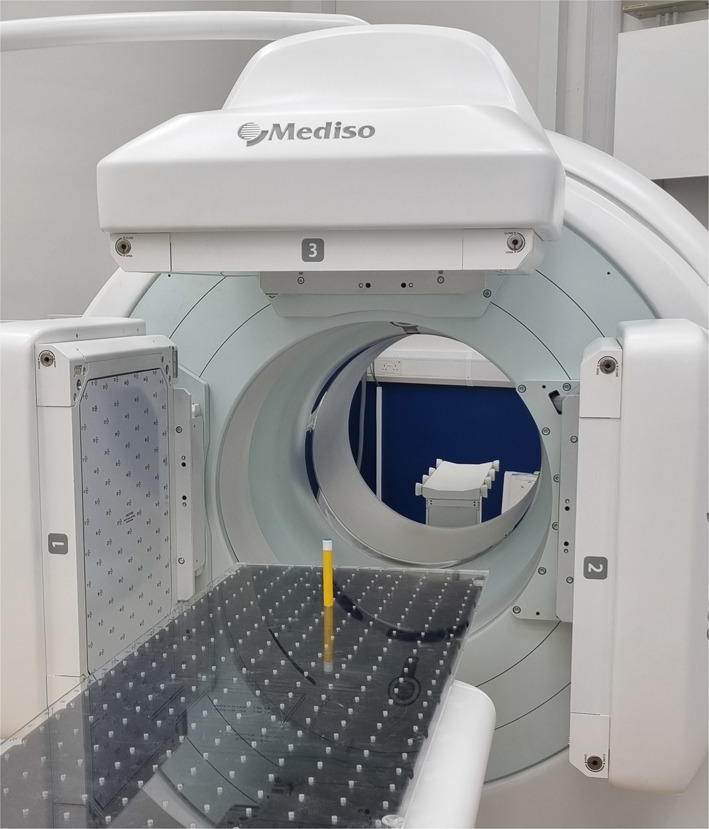
A photograph showing one of the calibration ampoules in a reproducible position on a custom-made perspex board beneath a stationary SPECT head of the Mediso AnyScan SPECT system. The same position was used for each source

Standard calibration sources were used to determine the experimental intrinsic efficiency of the Mediso SPECT AnyScan system, these were $^{109}$Cd, $^{133}$Ba, $^{139}$Ce, $^{210}$Pb, $^{241}$Am, $^{99\text {m}}$Tc, $^{123\text {m}}$Te, and $^{177}$Lu. A liquid solution of each radionuclide was dispensed to a 2 ml ISO ampoule [20] and placed in a reproducible position with the SPECT head at a fixed radius of 455 mm. No collimators were used. All calibration source data were acquired with a $^{99\text {m}}$Tc energy-correction map [21]. A photograph of the set-up is shown in Fig. 3. All sources were traceable to national primary activity standards, with activities as shown in Table 4. Static acquisitions were acquired for 600 s for each source, apart from $^{109}$Cd and $^{210}$Pb which were acquired for 3600 and 7200 s, respectively, to account for their low count rates.

**Table 4:** The $\upgamma$ emissions used to calculate the intrinsic detector efficiency

Isotope	Energy (keV)	Intensity (%)	Activity (kBq)
$^{210}$Pb	46	4.25 ± 0.04	32.82 ± 0.34
$^{241}$Am	60	35.9 ± 0.4	276.2 ± 1.0
$^{133}$Ba	81	32.9 ± 0.3	129.30 ± 0.88
$^{109}$Cd	88	3.644 ± 0.016	75.54 ± 0.46
$^{177}$Lu	113	6.23 ± 0.04	512.3 ± 2.5
$^{99\text {m}}$Tc	140	89 ± 4	170.6 ± 1.5
$^{123\text {m}}$Te	159	84.0 ± 0.4	58.325± 0.085
$^{139}$Ce	166	79.90 ± 0.04	50.75 ± 0.51
$^{177}$Lu	208	10.41 ± 0.04	512.3 ± 2.5
$^{133}$Ba	356	62.05 ± 0.19	129.30 ± 0.88

The quoted activities correspond to the start of the planar acquisition and standard uncertainties are given. $\upgamma$ emission intensities are taken from [22]. Energy uncertainties are not given as they were significantly smaller than the energy resolution of the SPECT camera

A Gaussian fit was applied to each photopeak in the energy spectrum of each isotope. An absolute detection efficiency was then calculated for each of the peaks according to

1 $$\begin{aligned} \epsilon = \frac{N}{A \times I_\gamma } \end{aligned}$$

where *N* is the net count rate in the peak, *A* is the source activity, and $I_\gamma$ is the emission intensity of the $\upgamma$ ray of interest. The uncertainty on the absolute efficiency, $\sigma _\epsilon$, was determined through standard uncertainty propagation, assuming uncorrelated variables, as

2 $$\begin{aligned} \sigma _\epsilon ^2 = \left| \frac{\partial \epsilon }{\partial N}\right| ^2 \sigma _N^2 + \left| \frac{\partial \epsilon }{\partial A}\right| ^2 \sigma _A^2 + \left| \frac{\partial \epsilon }{ \partial I_{\gamma } }\right| ^2 \sigma _{I_{\gamma }}^2 = \epsilon ^2 \left[ \left( \frac{\sigma _N}{N_{~}}\right) ^2 + \left( ~\frac{\sigma _A}{A_{~}}\right) ^2 + \left( \frac{\sigma _{I_{\gamma }}}{I_{\gamma }}\right) ^2 ~\right] \end{aligned}$$

where $\sigma _N$, $\sigma _A$ and $\sigma _{I_{\gamma }}$ are the uncertainties on the net count rate, activity and emission intensity, respectively.

### Simulation model

A full MC simulation model was created for the Mediso AnyScan SPECT system based on specifications provided by the manufacturer and physical measurements. GATE version 8.2 with Geant4 version 10.05 was used. A detector head model was created and positioned for either dual or triple head configuration, as per the physical camera. Each head contained a 9.5 mm NaI thick crystal set as a ‘sensitive volume’, meaning all interactions within the volume were recorded. Geometric structures for the glass light-guide and the compartment housing the signal electronics were modelled in the simulation, as they were found to contribute to scatter into the crystal (as seen in other work [23]). The individual photomultiplier tubes were not defined in the simulation; instead, a general ‘back-compartment’ material was used to mimic the density of the electrical components, as suggested in [3]. In addition to SPECT, the AnyScan SCP system has a computed tomography (CT) and a positron emission tomography (PET) ring, but these were not included in the simulation.

Models for LEHR and MLEGP collimators were defined as uniform blocks of lead with a rectangular array of hexagonal holes according to technical specifications of hole size and septal thickness, shown in Table 1. An aluminium touch-plate was included in front of the collimator and the detector head was surrounded by lead shielding. The plastic casing surrounding the heads was not included in the simulation. A visualisation of the simulation model with a geometric cylindrical phantom on the patient bed is shown in Fig. 1, along with a photograph of the physical scanner.

The patient bed was defined from a CT image, in order to accurately describe its internal structure.

The CT scan of the phantoms was used to define the source geometry and attenuation distribution in the simulation. The Hounsfield unit (HU) value of each voxel was attributed to a material density and elemental composition. The CT images were manually segmented to determine regions for activity. The CT scan was used to ensure accurate replication of the positioning and material composition of the phantom in the simulation. The ‘ImageNestedParameterisedVolume’ feature in GATE was used to speed-up the simulation of voxelised volumes [24]. For the calibration ampoules and point sources, geometric source and phantom distribution were used due to the more simple geometry and material composition.

#### Nuclear data, physics and digitisation models

Each radioactive source was defined as an isotropic ‘UserSpectrum’ with nuclear data on radioactive emissions, energies and half-life from the ENSDF database [22]. All emissions of $\upgamma$ rays and X-rays were included as a discrete energy histogram according to the energy and intensity provided by ENSDF. For $^{177}$Lu, the $\upbeta$ particle emissions were also included in the source definitions as an ‘Arb’ histogram with linear interpolation, so that any contributions from Bremsstrahlung were included. Radioactive decay was included in all simulated acquisitions. In each case, the full experimental activity was simulated.

All physics processes such as scatter and attenuation within the phantom, collimator and detector system were defined with the Geant4 *emstandard_opt4* library with its default parameters; this physics library has undergone extensive validation [25]. Production range cuts of 0.05 mm were applied to photon tracking and 0.01 mm to electron tracking in the simulation. These are converted into an energy threshold for each material, below which no secondary particles are generated.

The scintillation process and light detection were not incorporated into the simulation due to large computation times. Instead, the photon-detection process was modelled through GATE digitisation modules, which convert photon interactions in the crystal into digital counts and apply blurring to mimic the response of the physical system. An ‘Adder’ module was used to sum detected events. A Gaussian blurring of the energy spectrum was applied by with the ‘blurring’ command, defining an energy resolution of 9.5% at 140 keV, as stated by the manufacturer. The energy resolution was extrapolated to other energies using GATE’s in-built default inverse square law function. A Gaussian intrinsic spatial blurring was defined with GATE’s ‘spblurring’ with full-width half maximum of 3.2 mm as quoted by the manufacturer [15]. The timing resolution of the detector was not modelled as the dead-time percentage was found to be insignificant at the activities used in this work ($<0.05\%$ at 200 MBq of $^{99\text {m}}$Tc which is almost twice the phantom activities used here).

#### Simulation execution and post-processing

A simulation was conducted for each of the experimental acquisitions of point-sources, calibration ampoules and phantoms. All simulations were split into parallel jobs and run on a HTCondor cluster [26] with 660 total CPU cores.

The GATE ‘Singles’ output of all simulations were recorded in TTrees in ROOT, an object-oriented programme developed by the CERN community [27]. Simulated energy spectra were extracted directly from the TTrees.

**Fig. 4:**
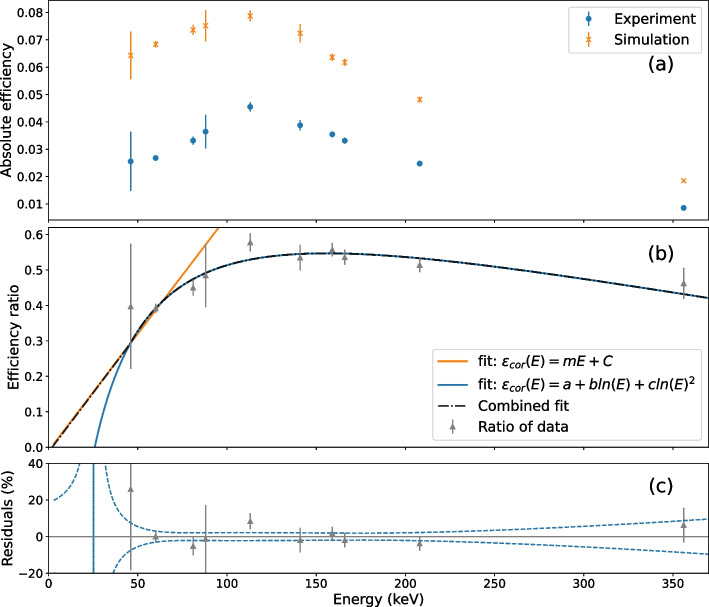
The data used to create the efficiency correction. **a** The absolute efficiency calculated from the experimental and simulated calibration sources. **b** The data points with the two fits as discussed in the text and the superposition of the two functions which was used for the efficiency correction. The points show the ratio of experimentally measured and simulated efficiency for the calibration sources with their standard uncertainties. **c** The residuals of the fit to the data. The error bars on the residuals are the standard uncertainty of each data point and the dashed lines represent the uncertainty of the logarithmic fit

To assess the intrinsic efficiency, a Gaussian fit was applied to the photopeaks in the simulated energy spectra of the calibration ampoules. Equation 1 was used to calculate the absolute detection efficiency of the simulation as a function of energy; these are compared to the same measurements from the experimental data in Fig. 4a. The intrinsic efficiency measurements showed that the simulation consistently overestimated the efficiency and this effect was worse at low energy, suggesting that the simulation digitisation modules do not accurately represent the signal-processing chain of electronics in the physical system. To correct for this, the ratio of the experimental and simulated efficiency was calculated for each peak of the calibration sources. An efficiency correction function, $\epsilon _{\text {cor}}$, was modelled as a function of energy, *E*, using the equation

3 $$\begin{aligned} \epsilon _{\text {cor}}(E) = a + b \text{ln}(E) + c \text{ln}^2(E) \end{aligned}$$

where *a*, *b* and *c* are the fitted parameters of the function and ln is the natural logarithm. Such a function is commonly used for the efficiency of NaI detectors [28]. The fitted $\epsilon _{\text {cor}}$ returned zero efficiency at 26 keV, rather than 2 keV seen in the experimental data. Thus, the efficiency was fixed to zero at 2 keV and a linear extrapolation was applied from the lowest-energy experimental data point. This energy region is significantly lower than energies typically used for clinical SPECT imaging, so the linear extrapolation was deemed sufficient. Figure 4b displays the logarithmic and linear functions applied to the efficiency-correction data. Residuals of $\epsilon _{\text {cor}}$ to the data above 46 keV (the lowest experimental data point) are shown in Fig. 4c; the error bars are the standard uncertainty on each data point (calculated with Eq. 2) and the dashed lines represent the uncertainty of the fit. The fit uncertainty was taken as the standard deviation of 1000 fits after perturbing each data point by a random number following a normal distribution with sigma equal to the point’s standard uncertainty.

A combination of the logarithmic and linear functions was used to correct the simulation efficiency:

4 $$\begin{aligned} \epsilon _{\text {cor}} = \left\{ \begin{array}{ll} - 0.0134 + 0.0067 ~ E &{} \quad \text{ if } E < 46~{ \text{ keV }}, \\ -3.755 + 1.702 ~\text{ln}(E) -0.168 ~\text{ln}^2(E) &{} \quad \text{ if } E \ge 46~\text { keV}. \end{array} \right. \end{aligned}$$

This function was applied to the simulation output through Poisson sampling [29] on an event-by-event basis to correct the efficiency of the simulation for all acquisitions. The experimental background energy spectra were time-corrected and added to the simulated energy spectra in ROOT. The manufacturer-provided software to extract the experimental energy spectra resulted in a slightly different number of counts compared to those from the projection images. The simulated energy spectra were therefore normalised to the same total counts as the experimental energy spectra for each phantom. This normalisation was applied only for the comparison of energy spectra and not in other analyses, so did not affect the sensitivity comparison.

To create projection images, ROOT was used to split each simulation into 120 projections to replicate each static position of a detector head and set gates for the relevant energy and scatter windows. An in-house code was then used to generate an interfile image for each projection, applying the same matrix and pixel sizes as the experimental acquisitions. The background projections with the relevant energy window were time-corrected (with Poisson sampling) and added to the simulated projections on a pixel-by-pixel basis.

### Validation metrics

Several experimental observables were identified and used to validate the MC simulation. These were the sensitivity, energy spectrum, spatial resolution, sinograms (counts per time frame), and projection images.

The spatial resolution of the system was measured using the planar acquisitions of a point source filled with $^{99\text {m}}$Tc in air. A linear profile with a 1-pixel width was defined through the centre of each simulated and experimental image, and the counts along that profile were recorded. The SciPy Orthogonal Distance Regression Python package [30] was used to apply Gaussian fits to each profile, accounting for uncertainty in both dimensions. The Poisson statistical uncertainty was used as the uncertainty on the counts for each pixel.

The other metrics were validated through tomographic phantom acquisitions of $^{99\text {m}}$Tc. Manufacturer-provided software was used to extract the energy spectrum from all acquisitions for a direct comparison to the simulation. Sinograms were used to validate that the movement of the SPECT heads was replicated in the simulation and that the phantom and source had been defined appropriately. The peak signal-to-noise ratio was used as quantification of the similarity of simulated and experimental images. Peak signal-to-noise ratio, PSNR, is defined for comparison of a simulated image *g* to an experimental image *f*, both of size $M \times N$, as

5 $$\begin{aligned} \text {PSNR} (f,g) = 20 \log _{10} \left\{ \frac{ \text {MAX}_f }{ \sqrt{ \text {MSE} } } \right\} \end{aligned}$$

where the mean squared error (MSE),

6 $$\begin{aligned} \text {MSE} = \frac{1}{MN} \sum _{i=0}^{M-1}\sum _{j=0}^{N-1} ( f(i,j) - g(i,j) )^2 \end{aligned}$$

and $\text {MAX}_f$ is the maximum possible pixel value of image *f* [31]; all images were unsigned 16-bit, so this value was 32768. The PSNR value tends to infinity as the mean square error decreases, hence larger values of PSNR imply more-similar images. Since PSNR is not an absolute quantity, a reference was created to provide a baseline against which to compare the PSNR of the simulation and experiment. The reference PSNR was calculated by comparing the experimental images to themselves shifted by 1 pixel in each horizontal axis. The PSNR was calculated independently for each image slice, and the weighting mean and standard deviation of all slices was computed.

The absolute sensitivity, *S*, was calculated for the $^{99\text {m}}$Tc and $^{177}$Lu phantom acquisitions from the counts in each experimental and simulated image as

7 $$\begin{aligned} S = \frac{\text {c}}{A \times T} \end{aligned}$$

where *c* is the total counts in the image, *A* is the source activity, and *T* is the total acquisition time. This sensitivity was specific to the isotope, not the single emission as both emission and scatter windows were considered. Therefore, the branching ratio of specific photon emissions was not included in the absolute sensitivity calculation. Since the efficiency correction was applied to the simulation, its uncertainty was incorporated into the uncertainty of the sensitivity. The average uncertainty on the efficiency correction within the relevant energy window, $\bar{\sigma }_{\epsilon _{\text {EW}}}$, was used. The uncertainty on the sensitivity, $\sigma _S$, was calculated through

8 $$\begin{aligned} \left( \frac{\sigma _S}{S}\right) ^2 = \left( \frac{\sigma _c}{c}\right) ^2 + \left( \frac{\sigma _A}{A}\right) ^2 + \left( \frac{\bar{\sigma }_{\epsilon _{\text {EW}}}}{\bar{\epsilon }_{\text {EW}}}\right) ^2 \end{aligned}$$

where $\sigma _c$ and $\sigma _A$ are the uncertainties on the counts and activity and the uncertainty on *T* is assumed to be negligible. For the simulation, the uncertainty on the activity was zero. The simulation uncertainty was dominated by the uncertainty on the efficiency-correction function; for the $^{99\text {m}}$Tc and $^{177}$Lu emission and scatter windows, $\frac{\bar{\sigma }_{\epsilon _{\text {EW}}}}{\bar{\epsilon }_{\text {EW}}}$ ranged from 2.0 to 4.4%. For the experimental images, the efficiency term was zero and the uncertainty was dominated by the uncertainty in activity (which was below 1% for both isotopes).

The uncertainties on experimental and simulated data were quantified in each step of the validation process. Standard uncertainties (k=1) are quoted throughout this work unless otherwise specified.

## Results

### Spatial resolution

**Fig. 5:**
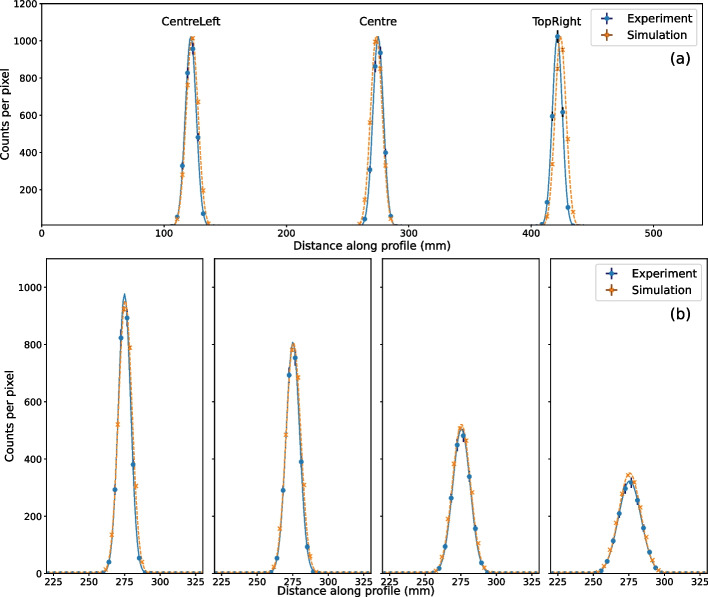
The counts in each pixel of a linear profile drawn through the centre of the simulated and experimental point-source images. The counts data are shown as points with standard uncertainties and the Gaussian fit applied to each data set is a solid line. **a** Varying lateral source position (maximum value normalised to average of the three). **b** Varying detector radius (no normalisation), the detector radius is given above each sub-plot

The simulated and experimental extrinsic spatial resolution for the LEHR collimators for a $^{99\text {m}}$Tc point source in air was compared for both the lateral and the vertical variations. Figure 5 shows the counts in each pixel along the profiles of the experimental and simulated data and the Gaussian fits to these data. The corresponding values of the full-width half maximum (FWHM) for these Gaussian fits are given in Table 5.

**Table 5:** The full-width half maximum (FWHM) of the Gaussian fits to each profile of the spatial resolution measurements

	FWHM (mm)	
	Experiment	Simulation
*Position name*		
Centre	10.14 ± 0.22	11.15 ± 0.15
CentreLeft	9.64 ± 0.20	10.64 ± 0.13
TopRight	10.50 ± 0.21	11.39 ± 0.12
*Detector radius* (mm)		
106.5	10.14 ± 0.22	11.15 ± 0.15
150.0	11.24 ± 0.23	12.13 ± 0.13
250.0	14.50 ± 0.29	15.05 ± 0.12
350.0	18.81 ± 0.51	18.38 ± 0.14

### Validation with $^{99\text {m}}$Tc phantoms

The following subsections present the results for validation against the experimental phantom measurements of $^{99\text {m}}$Tc with LEHR collimators.

#### Energy spectrum

**Fig. 6:**
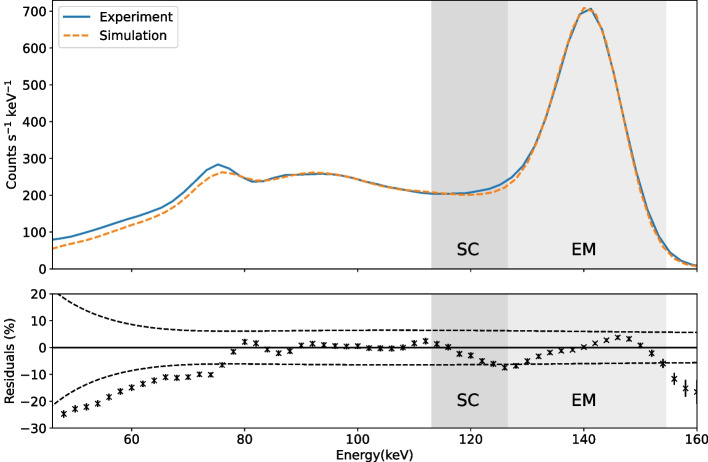
A comparison of the total simulated energy spectrum and the total spectrum extracted from the SPECT images for the cylindrical phantom of $^{99\text {m}}$Tc, plotted with 2 keV bins. The bottom plot shows the percentage residuals with standard uncertainties and the 3$\sigma$ bounds of the uncertainty on the efficiency correction shown as dashed lines. The emission (EM) and scatter (SC) windows are shaded

The simulated and experimental energy spectra were plotted in units of total counts per second per keV with 2 keV bins. Figure 6 shows the energy spectra for the cylindrical phantom of $^{99\text {m}}$Tc and the percentage residuals between the two spectra. The uncertainty on each data point in the residual plot is statistical and is taken as the square root of the total counts in that bin normalised to the scan time (which is assumed to have a negligible uncertainty). The standard deviation, $\upsigma$, of the efficiency-correction function was calculated for each energy bin. To quantify the agreement for the emission and scatter window energy ranges, a weighted mean of these residuals was calculated as (0.6 ± 1.3)% and ($-$4.5 ± 1.6)% for EM and SC, respectively.

#### Sensitivity

The sensitivity was calculated according to Eq. 7 for the simulated and experimental data for both $^{99\text {m}}$Tc acquisitions and is given in Table 6. A ratio of the simulated and experimental sensitivity was calculated. The dominant source of uncertainty in the experimental sensitivity is the activity uncertainty. The simulation uncertainty was greater and dominated by the efficiency-correction function; this uncertainty was energy-dependent so the average over each energy window was used.

**Table 6:** The sensitivity for the emission window (EM) and scatter window (SC) images of the $^{99\text {m}}$Tc phantoms with standard uncertainties

Isotope	Phantom	Window	Sensitivity (counts/(s$\times$MBq))		Sensitivity ratio
			Simulation	Experiment	(Sim/Exp)
$^{99\text {m}}$Tc	Cylinder	EM	36.33 ± 0.73	37.39 ± 0.33	0.972 ± 0.021
		SC	8.74 ± 0.19	7.865 ± 0.071	1.111 ± 0.026
$^{99\text {m}}$Tc	NEMA	EM	24.77 ± 0.50	27.06 ± 0.24	0.915 ± 0.020
		SC	4.464 ± 0.095	4.376 ± 0.039	1.020 ± 0.023

The last column gives the sensitivity ratio (simulation/experiment) which was used to normalise the simulation to validate projection and profile data

#### Sinograms and images

The acquisition of the NEMA phantom was used to validate the sinograms due to the greater variation in counts per projection compared to the cylindrical phantom.

**Fig. 7:**
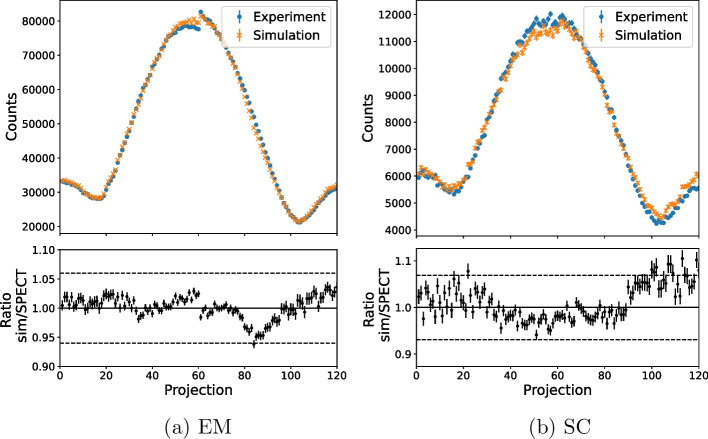
The counts per projection in the emission (EM) and scatter (SC) window of the simulated and experimental images of the NEMA phantom of $^{99\text {m}}$Tc. The simulation has been normalised using the sensitivity ratio given in Table 6. The bottom plots show the ratio of the simulated and SPECT counts for each projection and the dashed lines show the $\pm 3\upsigma$ uncertainty of the sensitivity ratio

The simulated images were normalised using the sensitivity ratio for the emission or scatter window given in Table 6, such that the sinograms and images could be validated independently from any discrepancies in sensitivity. Figure 7 compares the counts in each projection of the experimental and simulated emission and scatter images of the NEMA phantom of $^{99\text {m}}$Tc. The ratio of simulation and experimental projection counts is shown in the bottom plot, with a value of 1 showing an exact agreement. The dashed lines show the $\pm 3\upsigma$ uncertainty of the sensitivity ratio used to normalise the simulation. The weighted mean of the ratio over all slices was found to be $1.0001 \pm 0.0006$ for EM and $0.999 \pm 0.001$ for SC, both within one standard deviation of one. A discontinuity can be seen around projection 60 in these plots, particularly in the EM plot. This discontinuity is partly due to the decay of the phantom activity during the scan time as the two heads acquire data simultaneously, so projection 59 (head 1) is acquired around 30 min after projection 60 (head 2). The radioactive decay was modelled in the simulation, so this discontinuity can also be seen in the simulated data. However, the discontinuity is greater for the experimental data. This suggests that there may be a difference in experimental sensitivity between the two detector heads of the system. The sensitivity of the physical camera is normalised across each detector head but not between different heads, so a small difference in sensitivity may be present.

**Fig. 8:**
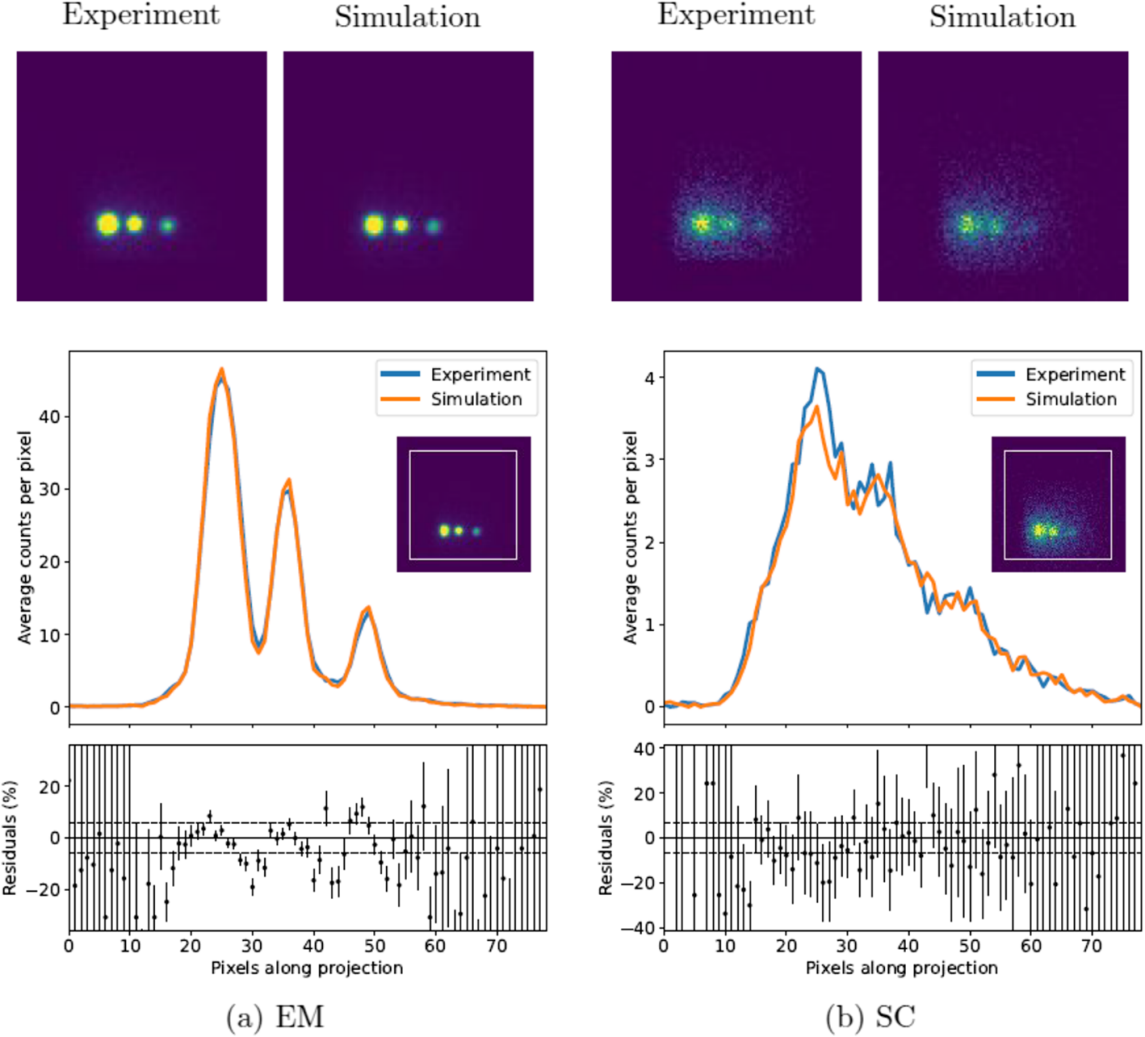
Top: experimental and simulated projection images for the NEMA phantom of $^{99\text {m}}$Tc for projection slice 38 is shown. The images have been cropped to 100$\times$100 pixels. Bottom: the vertically averaged counts along a rectangular profile (shown in white on the inset) of the simulated and experimental image. The percentage residuals are shown with the $\pm 3\upsigma$ uncertainty of the sensitivity ratio shown as dashed lines

The NEMA phantom was also used to verify whether the images themselves were comparable. Figure 8 shows a representative slice of the simulated and experimental images (projection 38 where the three largest spheres are visible). A square 80$\times$80 pixel profile was applied to the images and the vertically averaged counts along that profile are also compared in the figure. The peak signal-to-noise ratio was calculated for each slice of the experimental and simulated images, as described in Eq. 5, and a weighted mean was taken across all 120 slices. To put these numbers into context, the PSNR was also calculated by comparing the experimental image with itself following a shift of one pixel in each positive axis. The PSNR values are given in Table 7.

**Table 7:** The weighted mean of the peak signal to noise ratios (PSNRs) for each projection slice of the simulated and experimental images of the NEMA phantom of $^{99\text {m}}$Tc

Window	Simulation versus experiment (dB)	Reference- shifted experiment (dB)
EM	69.98 ± 0.34	67.13 ± 0.26
SC	88.00 ± 0.44	88.37 ± 0.50

The experimental image was shifted by one pixel in *x*- and *y*- axes as a reference for comparison

### Extending the validation to $^{177}$Lu phantoms

The same simulation model was used for the $^{177}$Lu data, to determine if the validation could be translated to another isotope and MLEGP collimators. The only change to the simulation was the energy resolution for $^{177}$Lu was set with an ‘energy blurring’ digitisation module of 8.7% at 208 keV (based on experimental measurements). Again the default inverse square function was used to propagate this energy blurring. The same efficiency-correction function which was measured with an $^{99\text {m}}$Tc energy correction map was applied.

#### Energy spectrum

**Fig. 9:**
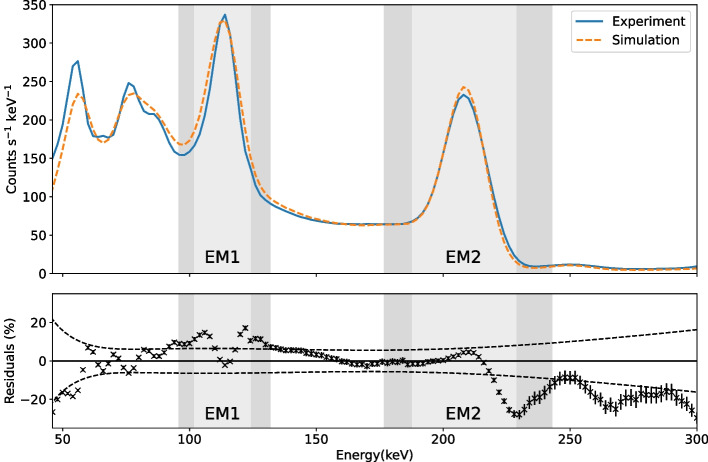
A comparison of the total simulated energy spectrum and the total spectrum extracted from the SPECT images for the cylindrical phantom of $^{177}$Lu, plotted with 2 keV bins. The bottom plot shows the percentage residuals of the simulation to the experimental spectrum with standard uncertainties. The emission windows and adjacent scatter windows are shaded. The 3$\sigma$ bounds of the uncertainty on the efficiency correction are shown as dashed lines

The simulated and experimental energy spectra were compared for the cylindrical phantom of $^{177}$Lu; these are displayed in Fig. 9. The percentage residuals were calculated and the weighted mean of these residuals was found to be $(6.20~\pm ~0.26)$% and $(-4.90~\pm ~0.22)$% for EM1 and EM2, respectively. Scatter windows SC1 and SC2, either side of EM1, had weighted means of $(9.22~\pm ~0.53)$% and $(10.22~\pm ~0.50)$%, respectively. For the scatter windows adjacent to EM2, the weighted means were $(-0.81~\pm ~0.41)$% for SC3 and $(-19.97~\pm ~0.37)$% for SC4.

#### Sensitivity

The sensitivities of the simulated and experimental acquisitions of $^{177}$Lu phantoms and their ratios are given in Table 8. These data show that the simulation is consistently under-estimating the sensitivity for $^{177}$Lu, and this effect is worse for EM2 and its scatter windows than EM1.

**Table 8:** The total counts normalised to acquisition time and phantom activity for the two emission window images (EM1 and EM2) of the $^{177}$Lu phantoms with standard uncertainties

Isotope	Phantom	Window	Sensitivity (counts/(s$\times$MBq))		Sensitivity ratio
			Simulation	Experiment	(Sim/Exp)
$^{177}$Lu	Cylinder	EM1	7.63 ± 0.16	8.632 ± 0.043	0.884 ± 0.020
		SC1	1.369 ± 0.049	1.647 ± 0.008	0.831 ± 0.030
		SC2	1.094 ± 0.042	1.160 ± 0.006	0.943 ± 0.037
		EM2	7.15 ± 0.17	8.664 ± 0.043	0.825 ± 0.020
		SC3	0.925 ± 0.040	1.201 ± 0.006	0.770 ± 0.034
		SC4	0.140 ± 0.006	0.226 ± 0.001	0.622 ± 0.026
$^{177}$Lu	NEMA	EM1	6.27 ± 0.14	6.889 ± 0.035	0.910 ± 0.020
		SC1	1.385 ± 0.05	1.655 ± 0.009	0.837 ± 0.031
		SC2	0.999 ± 0.039	1.077 ± 0.006	0.928 ± 0.036
		EM2	5.36 ± 0.13	6.275 ± 0.032	0.855 ± 0.021
		SC3	0.975 ± 0.043	1.179 ± 0.006	0.827 ± 0.037
		SC4	0.184 ± 0.008	0.197 ± 0.001	0.935 ± 0.039

The last column gives the sensitivity ratio (simulation/experiment) which was used to normalise the simulation for the sinogram and profile validation

#### Sinograms and images

**Fig. 10:**
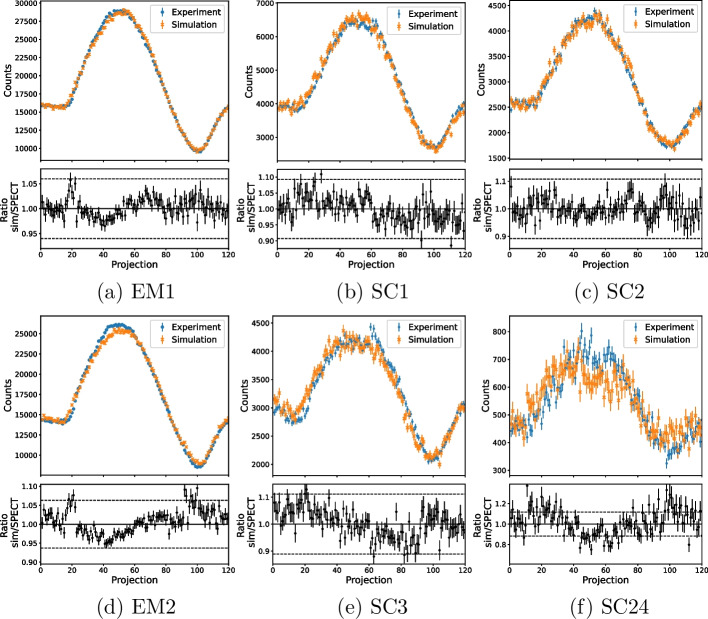
A comparison of the number of counts per projection in the emission windows of the simulation and the experimental SPECT images of the six-sphere NEMA phantom of $^{177}$Lu. The simulation has been normalised using the sensitivity ratio given in Table 8. Error bars show standard uncertainties. The bottom plots show the ratio of the simulated and SPECT counts for each projection. The dashed lines show the ±3$\upsigma$ uncertainty of the sensitivity ratio. A larger *y*-axis range is shown for the ratio of SC4

The six-sphere NEMA phantom was used to validate the $^{177}$Lu sinograms. Figure 10 shows the counts per projection of the simulated and experimental data. A weighted mean of the ratio for each projection slice was calculated for each data set. For the first energy window, these were (1.0001 ± 0.0012) for EM1 and (0.9982 ± 0.0025) and (0.9982 ± 0.0030) for SC1 and SC2, respectively. For the second, these were (0.9985 ± 0.0012) for EM2 and (0.9966 ± 0.0029) and (0.9795 ± 0.0068) for SC3 and SC4, respectively.

**Fig. 11:**
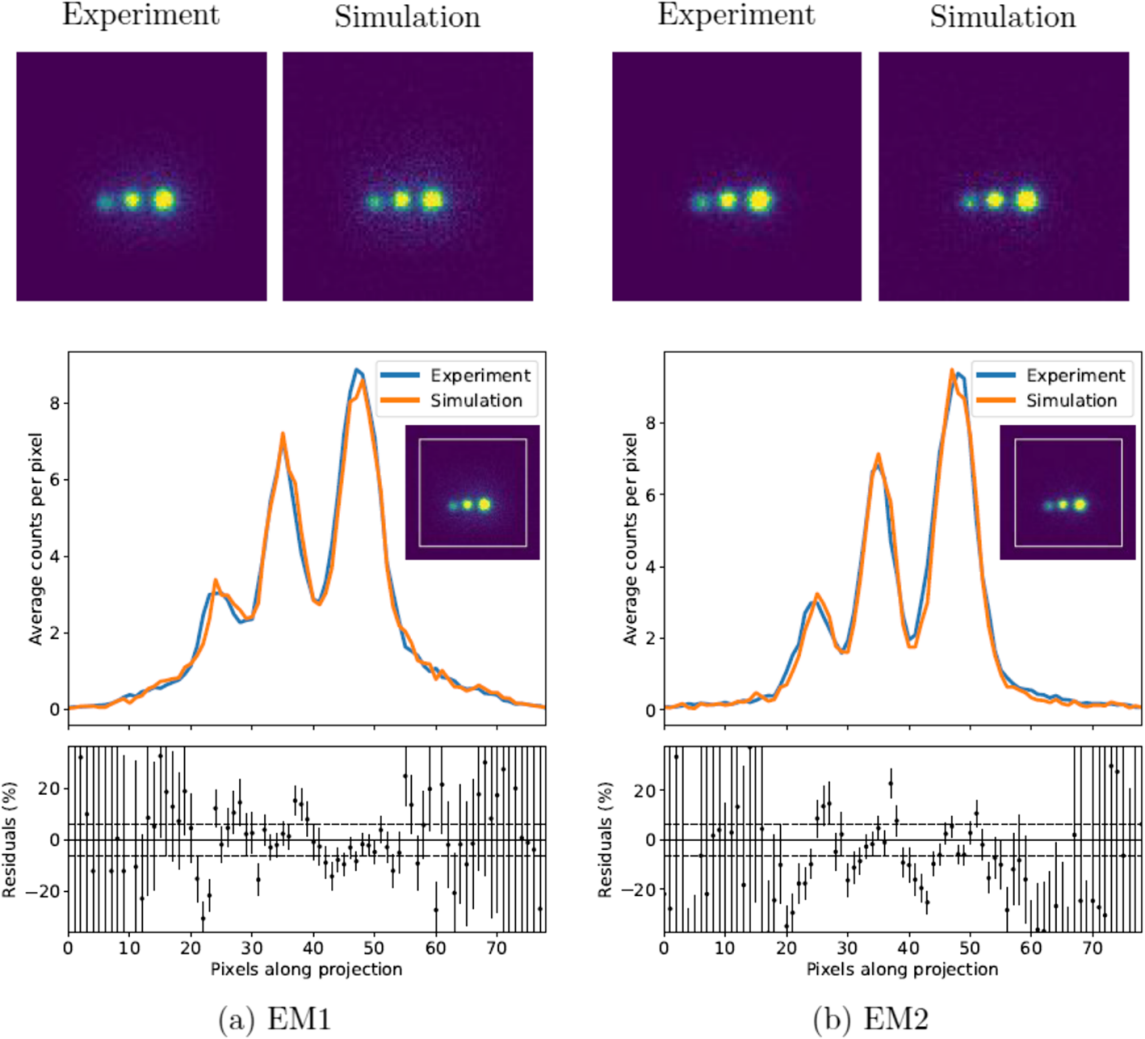
Top: experimental and simulated projection images for the NEMA phantom of $^{177}$Lu. Projection slice 48 is shown. The images have been cropped to 100$\times$100 pixels. Bottom: the vertically averaged counts along a rectangular profile (shown in white on the inset) of the simulated and experimental image. The percentage residuals are shown with the $\pm 3\upsigma$ uncertainty of the sensitivity ratio shown as dashed lines

The PSNR was calculated for only the emission windows of the $^{177}$Lu NEMA images, due to the high noise levels in the scatter images. A comparative slice of the experimental and simulated projection images is shown in Fig. 11, with the vertically averaged counts along a square profile. Again, a test image was created by shifting the experimental image by one pixel in the positive *x*- and *y*- axis; Table 9 shows that the PSNR of the simulated images compared to the experimental images is consistent with the PSNR of the shifted test image for both emission windows.

**Table 9:** The weighted mean of the peak signal to noise ratios (PSNRs) for each projection slice of the simulated and experimental images of the NEMA phantom of $^{77}$Lu

Window	Simulation versus experiment (dB)	Reference-shifted experiment (dB)
EM1	82.57 ± 0.49	81.07 ± 0.49
EM2	82.40 ± 0.60	79.43 ± 0.47

The experimental image was shifted by one pixel in *x*- and *y*- axes as a reference for comparison

## Discussion

### Spatial resolution

All experimental and simulated FWHM agree within three standard deviations (Table 5). There is close agreement in the Gaussian fits on the experimental and simulated profiles shown in Fig. 5. The simulation and experiment show an equivalent increase in FHWM with increasing distance between the source and detector. The Gaussian fits for the variation in lateral position also show agreement. The lateral positions were not changed by an integer number of pixels, therefore, despite the experimental images having an equivalent number of total counts, the maximum pixel value along the profile varied by 20% in the down-sampled images. The average maximum pixel value of the three acquisitions was used to normalise the data and fits. Here, the spatial resolution was only evaluated for $^{99m}$Tc; other work has investigated the energy-dependence of the extrinsic spatial resolution in GATE [32].

### Validation with $^{99\text {m}}$Tc phantoms

The simulated energy spectrum for the cylindrical phantom of $^{99\text {m}}$Tc, shown in Fig. 6, agrees well with experiment with weighted means of residuals consistent with zero for both the EM and SC windows. The suggests that any deviation is within the simulation’s uncertainty. Statistical agreement is lost below 76 keV. The measured efficiency correction had large uncertainty at low energy, mostly due to the low count rate from the $^{210}$Pb source. This energy range is below that typically considered in SPECT imaging, but further low-energy calibration points could be acquired if a more accurate low-energy efficiency correction was required, particularly below 46 keV where a linear extrapolation was used.

Perfect replication of the experimental sensitivity is particularly challenging as the electronic photon-detection chain is not modelled by the simulation. Furthermore, the simulation will always model a perfect system and will not contain defects in the crystal, collimators or other components which may be present in the experimental system. The sensitivity of the simulation depends heavily on the applied intrinsic efficiency correction. The efficiency-correction function used in this work had a maximum value of $\approx$0.5, showing that the simulation was overestimating the sensitivity by at least a factor of two before the correction was applied. For $^{99\text {m}}$Tc, the agreement between simulated and experimental sensitives is within 4$\upsigma$ in all cases. The efficiency was measured intrinsically, so it is also possible that differences in sensitivity are due to collimator manufacturing defects or slight misalignment in geometry. The calibration sources did not have sufficient activity to repeat the measurements with the collimators on the camera.

Figure 7 shows the agreement between the experimental and simulated sinograms. Any deviation between the experimental and simulated data is within the 3$\upsigma$ uncertainty bounds on the sensitivity ratio, suggesting that any discrepancy is within the 3$\upsigma$ uncertainty of the sensitivity correction. The weighted means of the relative counts per projection are within 1$\sigma$ of one for EM and SC, showing that the experimental and simulated sinograms are in close agreement.

The peak signal-to-noise ratios were comparable to or greater than those calculated by shifting the experimental image by one pixel in each positive axis. This suggests that the simulated and experimental images are at least as similar as the experimental images were with themselves following the shift.

### Extension to $^{177}$Lu phantoms

The validation method was repeated for phantoms of $^{177}$Lu. This isotope has a more complex emission spectrum than $^{99\text {m}}$Tc, so agreement was expected to be more challenging.

The comparison of the simulated and experimental energy spectra for $^{177}$Lu is shown in Fig. 9. There is some discrepancy, particularly below EM1, and the width of the EM1 peak appears to be wider in the simulation than experiment. (This is especially clear in the shape of the residuals in this area.) Other work has shown similar effects, where the intrinsic energy blurring in GATE does not produce agreement over the full energy spectrum of isotopes with multiple emissions. The simulated ^111^In spectrum was shown to agree better around the 171 keV peak than the 245 keV peak in [23], where the difference was attributed to the ‘distorted response of the gamma camera’ over the large energy range of emissions. Similarly, the energy spectrum around the 208 keV peak agreed better than the 113 keV peak for $^{177}$Lu in [33]. A different model for simulated energy spectra was proposed in [34]; this model may improve agreement over a large energy range but would have to be applied as a post-simulation correction outside GATE.

The sensitivity agreement for $^{177}$Lu was poorer than that seen for $^{99\text {m}}$Tc. The average sensitivity ratio for the two phantoms was (0.897 ± 0.014) for EM1 and (0.839 ± 0.014) for EM2, similar to a sensitivity difference of 20.1% seen in other work on GATE simulations of $^{177}$Lu acquisitions [35]. The calibration source data that was acquired to derive the efficiency correction (see Table 4) used the same $^{99\text {m}}$Tc energy-correction map as the phantom acquisitions. Subsequent $^{177}$Lu acquisitions were acquired using a different, $^{177}$Lu-specific, energy-correction map, which may alter the camera’s sensitivity. This may account for the simulation consistently underestimating the sensitivity for $^{177}$Lu. If other work required accurate simulated sensitivity for multiple isotopes, an efficiency correction function, such as Eq. 4, could be calculated for data acquired with each energy-correction map used in experimental work. In addition, the simulation assumes that the collimator is a uniform block of lead with a perfectly regular array of holes. This may not accurately reflect the physical collimator which could contain slight misalignments or non-uniformity in density due to manufacturing tolerances. Here, all simulated data were normalised by the simulation-experiment sensitivity ratio to ensure that other observables could be validated independently from any discrepancies in sensitivity.

Following the sensitivity normalisation, the simulated $^{177}$Lu images showed statistical agreement with experimental data. Figure 10 shows the sinograms agree closely, with all deviation within uncertainty on the sensitivity ratio and the weighted means all within 2 $\sigma$ of unity, with the exception of SC4 which is 3.0 $\sigma$ away from unity. The sinograms verify that the simulation is able to generate tomographic data comparable to the physical SPECT camera with both LEHR and MLEGP collimators. The peak-signal-to-noise ratios for EM1 and EM2 for $^{177}$Lu are both in agreement with the test image, further verifying that the simulation can generate images equivalent to those from the physical camera.

This work was limited to studies using fixed detector radii. For simulation studies of clinical protocols, it may be necessary to use non-circular orbits to mimic the auto-contouring setting of some clinical systems. A procedure for including non-circular orbits in GATE has been shown in other work, where a 2.6% difference for profiles acquired with circular and non-circular orbits was demonstrated for $^{177}$Lu [36]. Additional sinogram validation with non-circular orbits should be performed if non-circular orbits are required.

### Validation methodology and recommendations

The validation methodology presented in this work attempts to systematically identify experimental observables and acquire appropriate experimental data with uncertainties. These measurements are then reproduced in the MC simulation, allowing comparison with the experimental data. Where experimental data have been used to determine characteristics of the system to apply corrections to the MC simulation, for example, the intrinsic efficiency, then the uncertainties on these corrections have been assessed and propagated to the simulation data. Although agreement between the experimental data and simulation within uncertainties is desirable for all observables, the emphasis of the validation should be on a realistic assessment of the uncertainties, to allow the level of agreement to be understood. Understanding the limitations of a simulation can help ensure that it is applied appropriately and give confidence in the results.

This work demonstrated that the application of an energy-dependent efficiency correction led to agreement between the experimental and simulated sensitivity. Simulations which require accurate sensitivities, or applications which use isotopes with multiple emissions or large scatter windows will benefit from measuring an energy-dependent sensitivity function and applying it on an event-by-event basis. Special care should be taken for isotopes with low-energy emissions, where the experimental sensitivity is particularly sensitive. Here, the intrinsic efficiency measurement was performed with a $^{99\text {m}}$Tc energy map and was found to translate more poorly to experimental sensitivity measurements of $^{177}$Lu, suggesting that isotope-specific sensitivity measurements may be required for precise sensitivity validation.

It should be further emphasised that any validation is only valid for a specific snapshot of the system and simulation code, and that this validation methodology should be repeated when either change. In the specific case of MC simulation of a SPECT system this is particularly important when considering different radionuclides for which the precision of available nuclear data and interaction models can vary significantly. Likewise, many of the experimental observables such as sensitivity and intrinsic efficiency are dependent on measurement electronics and software meaning they may drift over time. Therefore, the validation method will need to be repeated if new updates are applied to the system. Ultimately, the necessary precision of a validation will depend on the specific applications for which the simulation is to be used, but this methodology can provide insight into quantification of the simulation’s uncertainty.

## Conclusion

It is essential to determine the validity of any simulation before it can be used to generate data. In this work, a Monte Carlo simulation of the Mediso AnyScan SCP system was created in the GATE simulation package and used to assess a quantitative validation methodology. The energy-dependent intrinsic efficiency was measured experimentally and used to create an efficiency-correction function which was applied to the simulation. The corrected simulation was then compared to several experimental observables to validate the simulation and quantify its agreement with the physical camera. A simulation will never perfectly replicate a physical system, particularly in nuclear medicine applications where full simulation of the photon-detection chain is generally too computationally expensive. This study was performed to quantify the similarity between the simulation and the physical camera. Energy spectra, planar and tomographic images were compared. Statistical agreement was seen in simulated and experiential images of both $^{99\text {m}}$Tc and $^{177}$Lu. A quantitative validation has now been established, so the simulation can be used with confidence for other applications such as energy window optimisation, scatter estimation and acquisition parameter testing. Furthermore, this quantitative validation methodology can be applied to other SPECT systems and MC packages.

## Data Availability

Simulation macros and data used during the current study are available from the corresponding author upon reasonable request.

## References

[1] Floyd CE, Jaszczak RJ, Harris CC, Coleman RE (1984). Energy and spatial distribution of multiple order Compton scatter in SPECT: a Monte Carlo investigation. Phys Med Biol.

[2] Ogawa K, Harata Y, Ichihara T, Kubo A, Hashimoto S (1991). A practical method for position-dependent Compton-scatter correction in single photon emission CT. IEEE Trans Med Imaging.

[3] Rault E, Staelens S, Van Holen R, De Beenhouwer J, Vandenberghe S (2011). Accurate Monte Carlo modelling of the back compartments of SPECT cameras. Phys Med Biol.

[4] Sohlberg AO, Kajaste MT (2012). Fast Monte Carlo-simulator with full collimator and detector response modelling for SPECT. Ann Nucl Med.

[5] Sarrut D, Bała M, Bardiès M, Bert J, Chauvin M, Chatzipapas K, Dupont M, Etxebeste A, Fanchon LM, Jan S (2021). Advanced Monte Carlo simulations of emission tomography imaging systems with gate. Phys Med Biol.

[6] Sarrut D, Baudier T, Borys D, Etxebeste A, Fuchs H, Gajewski J, Grevillot L, Jan S, Kagadis GC, Kang HG (2022). The OpenGATE ecosystem for Monte Carlo simulation in medical physics. Phys Med Biol.

[7] Ljungberg M, Strand S, King M. The SIMIND Monte Carlo program. In: Monte Carlo calculation in nuclear medicine: applications in diagnostic imaging (1998). p. 145–63.

[8] Augusto R, Bauer J, Bouhali O, Cuccagna C, Gianoli C, Kozłowska W, Ortega P, Tessonnier T, Toufique Y, Vlachoudis V (2018). An overview of recent developments in FLUKA pet tools. Phys Med.

[9] Solberg TD, DeMarco JJ, Chetty IJ, Mesa AV, Cagnon CH, Li AN, Mather KK, Medin PM, Arellano AR, Smathers JB (2001). A review of radiation dosimetry applications using the MCNP Monte Carlo code. Radiochim Acta.

[10] Harrison R, Haynor D, Gillispie S, Vannoy S, Kaplan M, Lewellen T (1993). A public-domain simulation system for emission tomography-photon tracking through heterogeneous attenuation using importance sampling. J Nucl Med.

[11] JCGM: JCGM 200: 2008 International vocabulary of metrology—basic and general concepts and associated terms ( VIM ) Vocabulaire international de métrologie - Concepts fondamentaux et généraux et termes associés ( VIM ). International Organization for Standardization Geneva ISBN 3(Vim) 104 (2008). 10.1016/0263-2241(85)90006-5. ISBN: 9282222136

[12] Jan S, Santin G, Strul D, Staelens S, Assié K, Autret D, Avner S, Barbier R, Bardiés M, Bloomfield PM, Brasse D, Breton V, Bruyndonckx P, Buvat I, Chatziioannou AF, Choi Y, Chung YH, Comtat C, Donnarieix D, Ferrer L, Glick SJ, Groiselle CJ, Guez D, Honore P-F, Kerhoas-Cavata S, Kirov AS, Kohli V, Koole M, Krieguer M, Laan DJVD, Lamare F, Largeron G, Lartizien C, Lazaro D, Maas MC, Maigne L, Mayet F, Melot F, Merheb C, Pennacchio E, Perez J, Pietrzyk U, Rannou FR, Rey M, Schaart DR, Schmidtlein CR, Simon L, Song TY, Vieira J-M, Visvikis D, Walle RVD, Wieërs E, Morel C (2004). GATE: a simulation toolkit for PET and SPECT. Phys Med Biol.

[13] Agostinelli S, Allison J, Amako K, Apostolakis J, Araujo H, Arce P, Asai M, Axen D, Banerjee S, Barrand G, Behner F, Bellagamba L, Boudreau J, Broglia L, Brunengo A, Burkhardt H, Chauvie S, Chuma J, Chytracek R, Cooperman G, Cosmo G, Degtyarenko P, Dell’Acqua A, Depaola G, Dietrich D, Enami R, Feliciello A, Ferguson C, Fesefeldt H, Folger G, Foppiano F, Forti A, Garelli S, Giani S, Giannitrapani R, Gibin D, Cadenas JJG, González I, Abril GG, Greeniaus G, Greiner W, Grichine V, Grossheim A, Guatelli S, Gumplinger P, Hamatsu R, Hashimoto K, Hasui H, Heikkinen A, Howard A, Ivanchenko V, Johnson A, Jones FW, Kallenbach J, Kanaya N, Kawabata M, Kawabata Y, Kawaguti M, Kelner S, Kent P, Kimura A, Kodama T, Kokoulin R, Kossov M, Kurashige H, Lamanna E, Lampén T, Lara V, Lefebure V, Lei F, Liendl M, Lockman W, Longo F, Magni S, Maire M, Medernach E, Minamimoto K, Freitas PMD, Morita Y, Murakami K, Nagamatu M, Nartallo R, Nieminen P, Nishimura T, Ohtsubo K, Okamura M, O’Neale S, Oohata Y, Paech K, Perl J, Pfeiffer A, Pia MG, Ranjard F, Rybin A, Sadilov S, Salvo ED, Santin G, Sasaki T, Savvas N, Sawada Y, Scherer S, Sei S, Sirotenko V, Smith D, Starkov N, Stoecker H, Sulkimo J, Takahata M, Tanaka S, Tcherniaev E, Tehrani ES, Tropeano M, Truscott P, Uno H, Urban L, Urban P, Verderi M, Walkden A, Wander W, Weber H, Wellisch JP, Wenaus T, Williams DC, Wright D, Yamada T, Yoshida H, Zschiesche D (2003). Geant4-a simulation toolkit. Nucl Instrum Methods Phys Res Sect A Accel Spectrom Detect Assoc Equip.

[14] Cajgfinger T, Rit S, Létang JM, Halty A, Sarrut D (2018). Fixed forced detection for fast SPECT Monte-Carlo simulation. Phys Med Biol.

[15] Mediso Medical Imaging Systems. AnyScan: triple modality molecular imaging system. https://pdf.medicalexpo.com/pdf/mediso/anyscan/94149-115577.html.

[16] Data Spectrum Corporation Products. http://www.spect.com/products-all.html. Accessed 27 Mar 2020.

[17] NEMA: National Electrical Manufacturers Association Standards Publication NU 2-2007, Performance Measurements of Positron Emission Tomographs (2007)

[18] Ljungberg M, Celler A, Konijnenberg MW, Eckerman KF, Dewaraja YK, Sjögreen-Gleisner K (2016). MIRD pamphlet no. 26: joint EANM/MIRD guidelines for quantitative 177lu SPECT applied for dosimetry of radiopharmaceutical therapy. J Nucl Med.

[19] Tran-Gia J, Denis-Bacelar AM, Ferreira KM, Robinson AP, Calvert N, Fenwick AJ, Finocchiaro D, Fioroni F, Grassi E, Heetun W (2021). A multicentre and multi-national evaluation of the accuracy of quantitative Lu-177 SPECT/CT imaging performed within the mrtdosimetry project. EJNMMI Phys.

[20] BS EN ISO 9187-2 (2010). Injection equipment for medical use. Part 2: one-point-cut (OPC) ampoules.

[21] Lawson R (2013). The gamma camera A comprehensive guide.

[22] From ENSDF database as of Dec 01 2022. https://www.nndc.bnl.gov/ensdf/.

[23] Assié K, Gardin I, Vera P, Buvat I (2005). Validation of the Monte Carlo simulator gate for indium-111 imaging. Phys Med Biol.

[24] Sarrut D, Guigues L (2008). Region-oriented CT image representation for reducing computing time of Monte Carlo simulations. Med Phys.

[25] Wenzel H, Yarba J, Dotti A (2015). The Geant4 physics validation repository. J Phys Conf Ser.

[26] Thain D, Tannenbaum T, Livny M (2005). Distributed computing in practice: the condor experience. Concurr Pract Exp.

[27] Brun R, Rademakers F (1997). Root-an object oriented data analysis framework. Nucl Instrum Methods Phys Res Sect A.

[28] Knoll GF (2010). Radiation detection and measurement.

[29] White D, Lawson RS (2015). A Poisson resampling method for simulating reduced counts in nuclear medicine images. Phys Med Biol.

[30] Virtanen P, Gommers R, Oliphant TE, Haberland M, Reddy T, Cournapeau D, Burovski E, Peterson P, Weckesser W, Bright J, van der Walt SJ, Brett M, Wilson J, Millman KJ, Mayorov N, Nelson ARJ, Jones E, Kern R, Larson E, Carey CJ, Polat I, Feng Y, Moore EW, VanderPlas J, Laxalde D, Perktold J, Cimrman R, Henriksen I, Quintero EA, Harris CR, Archibald AM, Ribeiro AH, Pedregosa F, van Mulbregt P, SciPy 1.0 Contributors (2020). SciPy 1.0: fundamental algorithms for scientific computing in Python. Nat Methods.

[31] Yuanji W, Jianhua L, Yi L, Yao F, Qinzhong J. Image quality evaluation based on image weighted separating block peak signal to noise ratio. In: International conference on neural networks and signal processing, 2003. Proceedings of the 2003, vol 2 (2003). p. 994–72. 10.1109/ICNNSP.2003.1281036.

[32] Holstensson M, Partridge M, Buckley SE, Flux GD (2010). The effect of energy and source location on gamma camera intrinsic and extrinsic spatial resolution: an experimental and Monte Carlo study. Phys Med Biol.

[33] Robinson AP, Tipping J, Cullen DM, Hamilton D (2016). The influence of triple energy window scatter correction on activity quantification for 177Lu molecular radiotherapy. Phys Med Biol.

[34] Morphis M, van Staden JA, du Raan H, Ljungberg M (2021). Modelling of energy-dependent spectral resolution for SPECT Monte Carlo simulations using SIMIND. Heliyon.

[35] Kayal G, Chauvin M, Vergara-Gil A, Clayton N, Ferrer L, Moalosi T, Knoll P, Struelens L, Bardiés M (2021). Generation of clinical 177Lu SPECT/CT images based on Monte Carlo simulation with gate. Phys Med.

[36] Kayal G, Chauvin M, Mora-Ramirez E, Clayton N, Vergara-Gil A, Tran-Gia J, Lassmann M, Calvert N, Tipping J, Struelens L (2022). Modelling SPECT auto-contouring acquisitions for 177Lu & 13Li molecular radiotherapy using new developments in geant4/gate. Phys Med.

